# Hemichannels; from the molecule to the function

**DOI:** 10.3389/fphys.2014.00411

**Published:** 2014-10-20

**Authors:** Mauricio A. Retamal, Juan C. Sáez

**Affiliations:** ^1^Centro de Fisiología Celular e Integrativa, Clínica Alemana Universidad del DesarrolloSantiago, Chile; ^2^Departamento de Fisiología, Pontificia Universidad Católica de ChileSantiago, Chile

**Keywords:** hemichannels, connexins, gap junction channels, pannexins, redox regulation, posttranslational modifications

## Introduction

Coordinated cell interactions are required to accomplish diverse complex and dynamic tasks of several tissues in vertebrates and invertebrates. Cell functions, such as intercellular propagation of calcium waves and spread of electrotonic potentials, are coordinated by cell-to-cell communication through gap junction channels (GJCs). These channels are formed by the serial docking of two hemichannels (HCs), which in vertebrates are formed by six protein subunits called connexins (Cxs). In humans, a gene family encodes 21 different proteins with a highly variable C-terminal where most posttranslational modifications occur. Among them protein phosphorylation and/or oxidation (e.g., nitrosylation) induces functional changes.

Currently, it is believed that undocked HCs may have functional relevance in cell physiology allowing diffusional exchange of ions and small molecules between intra- and extra-cellular compartments. In support to this new concept, it has been shown that controlled HC opening allows the release of small signaling molecules (e.g., ATP, glutamate, NAD^+^, adenosine, and cyclic nucleotides) and uptake of metabolically relevant molecules (e.g., glucose). Additionally, a growing body of evidences shows that HCs are involved in important and diverse processes, such PGE_2_ release from osteocytes, glucose detection in tanicytes, T cell infection with AIDS virus, memory consolidation in the basolateral amygdala and release of nitric oxide from endothelial cells, among others. However, HCs can also play an important role in the homeostatic imbalance observed in diverse diseases. In fact, enhanced HCs opening induces or accelerates cell death in several pathological conditions. Hemichannel-mediated cell death is due mainly to Ca^+2^ influx and cellular overload. The latter activates proteases, nucleases and lipases, causing irreversible cell damage. Accordingly, blockade of HCs reduces the cellular damage observed in several animal models of human diseases. Additionally, another family of proteins called pannexins (Panxs) also forms channels at the plasma membrane and some of their functional and pharmacological sensitivities overlap with those of Cx HCs. Recently, Panx channels have been involved in both pathological and physiological processes. Therefore, Cx HCs and Panx channels appear as promising drug targets for clinical treatment of several inherited and acquired human diseases.

This research topic gathers 11 articles that give a broad view about the role of Cx- and Panx–based channels from purified molecules reconstituted in a lipid environment and posttranslational regulation, to physiological and pathological implications. In addition, it proposes a putative molecular explanation of HC malfunctioning in specific diseases.

### Conflict of interest statement

The authors declare that the research was conducted in the absence of any commercial or financial relationships that could be construed as a potential conflict of interest.

